# Application of Mammalian Nudix Enzymes to Capped RNA Analysis

**DOI:** 10.3390/ph17091195

**Published:** 2024-09-11

**Authors:** Maciej Lukaszewicz

**Affiliations:** Department of Biophysics, Faculty of Physics, University of Warsaw, Pasteura 5, 02-093 Warsaw, Poland; maciej.lukaszewicz@fuw.edu.pl; Tel.: +48-22-5532353

**Keywords:** mRNA cap, synthetic cap analogues, NUDIX family, Nudt16, Nudt12, Nudt5, Nudt2, mRNA therapeutics

## Abstract

Following the success of mRNA vaccines against COVID-19, mRNA-based therapeutics have now become a great interest and potential. The development of this approach has been preceded by studies of modifications found on mRNA ribonucleotides that influence the stability, translation and immunogenicity of this molecule. The 5′ cap of eukaryotic mRNA plays a critical role in these cellular functions and is thus the focus of intensive chemical modifications to affect the biological properties of in vitro-prepared mRNA. Enzymatic removal of the 5′ cap affects the stability of mRNA in vivo. The NUDIX hydrolase Dcp2 was identified as the first eukaryotic decapping enzyme and is routinely used to analyse the synthetic cap at the 5′ end of RNA. Here we highlight three additional NUDIX enzymes with known decapping activity, namely Nudt2, Nudt12 and Nudt16. These enzymes possess a different and some overlapping activity towards numerous 5′ RNA cap structures, including non-canonical and chemically modified ones. Therefore, they appear as potent tools for comprehensive in vitro characterisation of capped RNA transcripts, with special focus on synthetic RNAs with therapeutic activity.

## 1. Introduction

mRNA-based technology has recently come into focus due to the development and worldwide use of new-generation vaccines against SARS-CoV-2 [1,2]. In the pioneering work, it was shown that in-vitro-synthesized mRNA supports protein synthesis after direct injection into skeletal muscle [3]. Thus, in addition to mRNA-based vaccines against infectious diseases, mRNA appears as a promising platform for other medically relevant applications, such as cancer immunotherapies or protein-replacement therapies [4]. This is highlighted by the 2023 Nobel Prize awarded to Katalin Kariko and Drew Weissman for their research on nucleoside base modifications of mRNA [5] and the long-standing research of other pioneers at the early stages of mRNA characterization [6].

**Figure 1:**
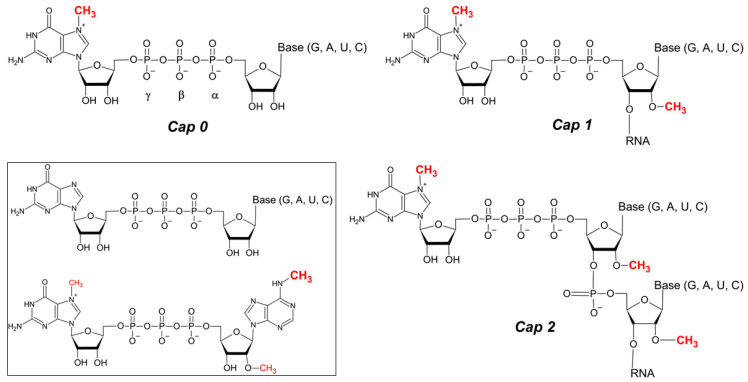
mRNA 5′ end cap structures. Methylations of m^7^Guo and at 2′O position of riboses of the first two transcribed nucleotides, which result in Cap 1 and Cap 2 structures, are indicated. Inset the unmethylated cap variant, and m^6^A_m_ adenosine containing cap variant (m^7^Gppp m^6^A_m_) are shown. Structures were drawn with ACD/ChemSketch, version 2022.1.2, Advanced Chemistry Development, Inc. (ACD/Labs).

In addition to the diverse modifications present on the eukaryotic mRNA chain [7], the 5′ end of mRNA end bears a specific cap structure, comprising of 7-methylguanosine (m^7^Guo) connected via 5′-5′ triphosphate bond to the first transcribed nucleotide of mRNA chain (m^7^GpppN, or Cap 0) [8] (Figure 1). Due to the biological roles of the 5′ cap in mRNA cellular functions (translation, stability, immunogenicity), it is a “hot-spot” for designing and synthesising chemically modified cap-structure analogues (e.g., [9,10]). For instance, the modified cap m_2_^7,3′O^GpppA_m_pG is already part of modified mRNAs in designed and distributed vaccine [1].

These chemically modified and synthesized cap analogues, once incorporated into mRNA, require characterisation in terms of capping efficiency, proper cap orientation, support of efficient cap-dependent translation, induction of cellular immunogenic response, and stability to enzymatic decapping. In this review, we focus on selected mammalian decapping enzymes of the NUDIX protein family, namely Dcp2, Nudt16, Nudt12, Nudt2 and Nudt5. These enzymes have been shown to act on distinct RNA cap structures in vivo and/or in vitro. Dcp2 and Nudt16, due to their initial discovery in the 5′ cap processing of mRNA and snRNA/snoRNA [11,12,13,14,15], have mostly been used to date for the detailed characterization and validation of mRNA transcripts with chemically modified 5′ caps. Including a wider set of Nudt decapping proteins with different or overlapping specificities towards chemically modified cap structures could be beneficial for a comprehensive understanding of the susceptibility to enzymatic degradation of in vitro designed RNAs. Furthermore, the use of human enzymes provides a more physiologically relevant environment for the analysis of modified caps, which is pivotal for the design, optimisation, and translation of therapeutic (m)RNAs into the clinic.

## 2. Diversity of 5′ RNA Cap Structures—Natural Caps

Beyond the aforementioned Cap 0 structure, the 5′ mRNA cap can be considered an extended structure where the first transcribed RNA nucleotides (namely N_1_ and N_2_ in the m^7^GpppN_1_pN_2_ structure) are 2′*O*-methylated, resulting in the m^7^Gpppm^2′^*^O^*N_1_ (Cap 1) or m^7^Gpppm^2′^*^O^*N_1_^2′^*^O^*N_2_ (Cap 2) structures [16]. Cap 0 is generally found in lower eukaryotes, whereas Cap 1 and Cap 2 are hallmarks of higher eukaryotes, including humans [8]. The most extensively methylated cap, Cap 4, has been identified in Kinetoplastida (e.g., *Typanosoma brucei*, *Trypanosoma cruzi* and *Leishmania major*), where the first four transcribed nucleotides adjacent to m^7^Guo (m^7^GpppN_1_pN_2_N_3_pN_4_) are heavily methylated (m^7^Gpppm_3_^6,6,2′O^Apm^2′O^Apm^2′O^C pm_2_^3,2′O^U) [17]. Additionally, adenosine as the first transcribed nucleotide of Cap 1 can be also methylated at the N^6^ position of the purine ring (m^6^A) both in the absence or presence of the 2′*O*-methylation. This modification has been shown to have a positive effect on translation efficiency in vitro [18] and on mRNA stability due to increased resistance to Dcp2 [19].

Eukaryotic RNA (e.g., snRNA, snoRNA, primary miRNA, pre-tRNA) and specific mRNAs (selenoprotein encoding transcripts, or *junD*, *dhx9* and *tgs1* mRNA) are modified at their 5′ ends with other cap structures, such as: trimethylguanosine cap (m_3_^2,2,7^GpppN), 5′-gamma-methyl (tri)phosphate cap (CH_3_pppN), and alfa-dimethyl monophosphate cap [20,21,22,23,24]. Additional 5′ cap structures have been detected on short RNAs (~20–200 nt), including non-methylated caps (GpppG and GpppA/ApppG), and next to the m^7^Gp or m_3_^2,2,7^Gp moiety, the dimethylguanosine (m_2_^2,7^Gp and m_2_^2,2^Gp), monomethylguanosine (m1Gp, m2Gp), and monomethylcitidine (mCp) structures. Moreover, the 2′*O*-methylation of the first transcribed nucleotide (N_1_) has been detected together with the aforementioned structures and trimethylated m_3_^2,2,7^Gp also with dimethyladenosine m^6^A_m_ [25,26], Table 1.

**Figure 2:**
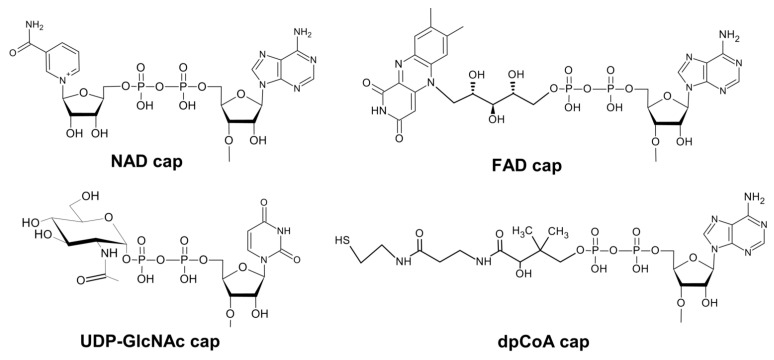
Structures of non-canonical RNA caps.

Although initially identified on bacterial RNAs [27], the diversity of naturally occurring 5′ caps present on mRNA and other types of eukaryotic cellular RNAs is further expanded by the detection of structures where the cap is composed of a non-canonical initiating nucleotide (NCIN): nicotinamide adenine dinucleotide NAD+ (NAD-cap) [28,29], flavin adenine dinucleotide cap (FAD-cap), dephospho-CoA (dpCoA-cap) or UDP-glucose derived caps (uridine diphosphate–glucose UDP-Glc cap and UDP-N-acetyloglucosoamie UDP-GlcNAc), so called “metabolic” cap structures (Figure 2) [30,31,32]. A very recent report showed that adenosine dinucleotide tetraphosphate (AppppA) can also serve as yet another type of non-canonical RNA cap (Ap_4_A-RNA) in eukaryotic cells (HEK293T, RBL-2H3 cell lines) [33].

**Table 1:** Diversity of naturally occurring cap structures on Eukaryotic cellular RNAs.

Cap Structure	RNA Type	Cell Type	Ref.
m^7^GpppN_1_ m^7^Gpppm^2′*O*^N_1_ m^7^Gpppm^6,2′*O*^A m^7^Gpppm^2′*O*^N_1_^2′*O*^N_2_	mRNA	Eukaryotic	[8,19,20]
m_3_^2,2,7^GpppN	snRNA (U1, U2, U4, U5), snoRNA primary miRNA (pri-miRNA) selenoprotein encoded transcripts, *junD*, *dhx9* and *tgs1* mRNA	Eukaryotic HPFs (human foreskin fibroblasts) HEK293, OSCA-40 canine osteosarcoma, HEK293FT	[20] [34] [22,23,24]
CH_3_pppN 5′-gamma-methyl (tri)phosphate cap	U6 and 7SK snRNAs	Eukaryotic	[35,36]
(CH_3_)_2_pN Alfa-dimethyl-phosphate cap	Precursor RNA of miR-145 (pre-miR-145)	MCF-7 and MDA-MB-231 breast cancer cells	[21]
m^7^GpppA(G) m_2_^2,7^GpppA(G) m_3_^2,2,7^GpppA(G)	pre-tRNA	*S. cerevisiae* HeLa	[25]
m^7^GpppA(G) mGpppC GpppG, GpppA m_3_^2,2,7^Gp, m_2_^2,7^Gp, m_2_^2,2^Gp m1Gp, m2Gp, mCp	Fraction of short RNAs (~20–200 nt), obtained from total RNA preparation	THP-1 (acute monocyte leukemia cell line)	[26]
NAD^+^ (5′ nicotinamide-adenine dinucleotide cap)	mRNA Intronic snoRNA mRNA	*S. cerevisiae* HEK293T Human (CCRF-SB cell line), mouse (C57BL/6 tissues), *S.cervisiae*	[28] [29] [37]
FAD	mRNA	Human (CCRF-SB cell line), mouse (C57BL/6 tissues), *S.cervisiae*	[37]
dpCoA	Mouse liver RNA	Mouse liver RNA	[32]
AppppA	RNA (>200 nt)	HEK293T RBL-2H3 (rat basophilic leukemia cells)	[33]
UDP-GlcNAc UDP-Glc	mRNA	Human (CCRF-SB cell line), mouse (C57BL/6 tissues), *S.cervisiae*	[37]

## 3. Diversity of Synthetic Cap Structures

Synthetic cap analogues, and synthetic capped (m)RNAs have proven to be invaluable tools for studying various cellular processes where the cap plays a pivotal role, including mRNA translation, splicing, intracellular transport, and RNA decay [38]. They are also important in studying cap-binding proteins and enzymes, including potential inhibitors of translation initiation factor eIF4E overexpressed in certain human malignancies and cancers (e.g., breast, colon, prostate, neck cancers) [39,40,41]. Now, following pioneering work demonstrating the first successful example of protein expression in muscle tissue after injection of in-vitro-transcribed capped mRNA [3] and the recent launch of anti-COVID-19 vaccines [1,2], capped RNAs have emerged as potent therapeutic agents with almost unlimited potential.

**Figure 3:**
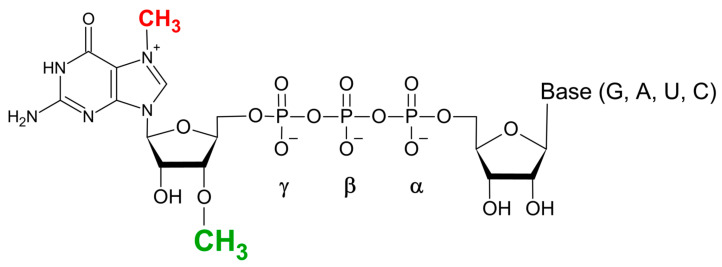
Anti-reverse cap analogue (ARCA).

In vitro transcription (IVT) methodology is a convenient approach for producing mRNA transcripts from a provided DNA template, allowing for co-transcriptional capping with synthetic dinucleotide cap analogues, resulting in capped-mRNA within a single reaction [42,43,44,45]. One of the problems with the direct using IVT-capped transcripts in therapeutic applications is the heterogeneity of the 5′ ends of the transcription reaction products, resulting in both m^7^GpppG-capped and reversely oriented Gppp(m^7^G)-capped transcripts due to priming of the polymerisation reaction by bacteriophage RNA polymerases (T7, SP6) from both ends of the m^7^GpppG cap dinucleotide [46]. The breakthrough in this issue was the development of anti-reverse cap analogues (ARCAs) by the Darzykiewicz lab, where the 3′-OH group of 7-methyl-guanosine (m^7^G) was either replaced by methyl group (m_2_^7,3′O^GpppG) (Figure 3) or removed (in 7-methyl-(3′deoxy)GpppG analogue) [47]. Such modifications prevent RNA polymerase from initiating from m^7^G moiety, resulting in the synthesis of transcripts capped exclusively in correct orientation, and thus in biologically active form. Similar ARCA-type properties have also been shown for dinucleotide cap analogues with 2′-OH methylated 7-methyl-guanosine [48].

**Figure 4:**
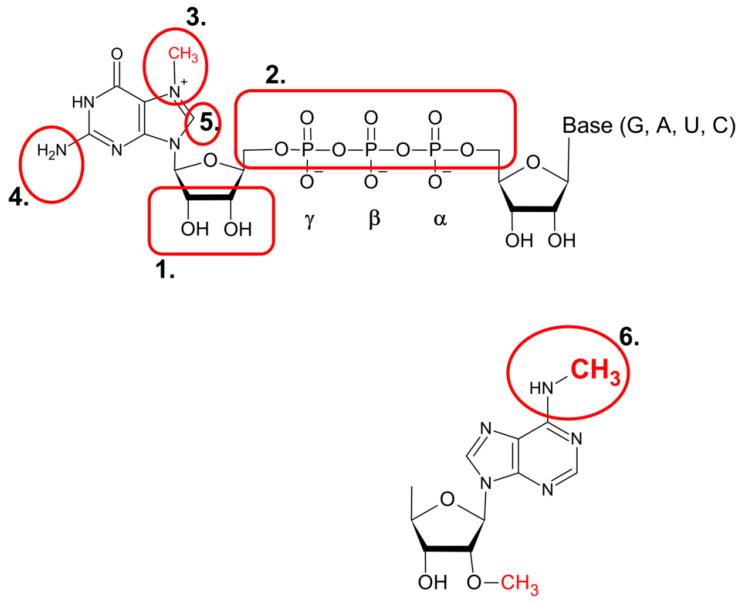
Sites of chemical modifications of the canonical cap structures. (**1**) 2′OH and 3′OH of m^7^G, (**2**) the triphosphate bridge, (**3**–**5**) substitutions at the N^7^, N^2^, and C8 position of guanine, respectively, (**6**) modifications at N^6^ position of adenine adjacent to the m^7^G in the cap nucleotide structure.

A natural choice for further chemical modifications was the triphosphate bridge of the dinucleotide cap structure (Figure 4), as it may protect the resulting analogues against decapping enzyme (e.g., Dcp2, [11,12,13,14]). Additionally, elongation of the triphosphate bridge to tetra- or pentaphosphate increases binding affinity towards eIF4E and has a positive effect on cap-dependent translation in vitro [48,49,50].

**Figure 5:**
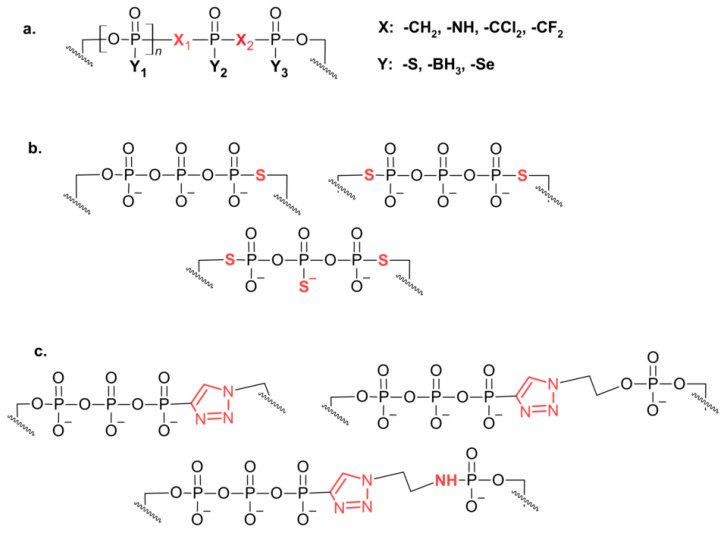
Examples of the modifications introduced into triphosphate bridge of mRNA cap dinucleotide. (**a**) X_1_, X_2_ indicate sites of bridging oxygen substitution with -NH, -CH_2_, -CCl_2_, or -CF_2_, and Y_1_, Y_2_, and Y_3_ indicate sites of non-bridging oxygen substitution with -S, -Se, or-BH_3_; (**b**) representative phosphorothiolate modifications after O-to-S substitution within 5′-phosphoester bond in the triphosphate chain; (**c**) examples of triazole moiety introduced within cap oligophosphate linkage.

Numerous different substituents have been used in synthetic cap analogs where at least one oxygen atom in the triphosphate bride was replaced, either the non-bridging α, β or γ oxygen (e.g., O-to-S, O-to-Se, O-to-BH_3_) [51,52,53,54,55,56], or bridging α-β and β-γ oxygen (e.g., O to NH, O to CH_2_, and O to CCl_2_, to CH_2_ or to CF_2_ in tetraphosphate analogs) [56,57,58,59]. In addition to cap analogues with a single replacement within the tri- or tetra-phosphate bridge, a series of multiple-modified compounds were synthesized, e.g., with double thiophosphate or double boranophosphate moieties, with or without O to CH_2_ exchange [60,61]. Another site of O-to-S substitution was the 5′-phosphoester bond in the triphosphate chain, resulting in a cap dinucleotide analogues with a phosphorothiolate moiety [62]. By applying a “click-chemistry” approach, a series of cap analogues containing the triazole moiety within the oligophosphate chain (with accompanying oxygen substitutions mentioned above in the text) were obtained, expanding the library of diverse substituents for the triphosphate bride modification [63,64,65] (Figure 5).

**Figure 6:**
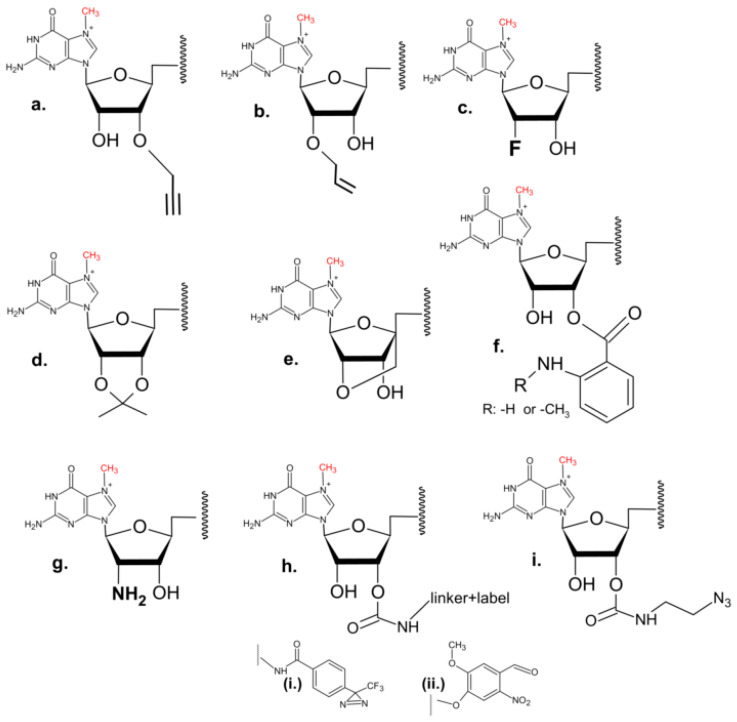
Examples of the modifications introduced at 2′-OH or 3′-OH of m^7^Guanine. (**a**) propagyl group; (**b**) allyl group; (**c**) 2′-OH to Fluor substitution; (**d**) isopropylidene group; (**e**) locked nucleic acid (LNA) modification; (**f**) anthraniolyl (R: -H) and methylanthraniolyl (R: -CH_3_) fluorescent moieties; (**g**) 2′ amino group; (**h**) example of carbamate modification, possible label moieties include: trifluoromethyl diazirine (**i**), 6-nitroveratryl alcohol (NVA) (**ii**), fluorescein or biotin; (**i**) example of modification with azide group.

Further modifications that utilize chemical modifications of either the 2′-OH or/and 3′-OH on m^7^G, similar to ARCA cap analogues, have also been reported. These include substitution of 2′-OH with a fluorine or allyl moiety [66,67], 2′-propagyl substituted di- and tri-nucleotide cap analogues [68,69], isopropylidene analogues [70,71], anthraniolyl and methylanthraniolyl fluorescent cap analogues [72], and locked nucleic acid (LNA) di- and tri-nucleotide cap analogues [73,74]. Analogues with 2′-amino groups instead of 2′-OH of m^7^G were next subject for biotin attachment, facilitating the capture of mRNA–protein complexes [75,76]. Further development of this series of cap analogues resulted in compounds carrying amino-functionalized linkers at 2′-OH or 3′-OH of m^7^G, allowing subsequent attachment of fluorescent moieties (e.g., fluorescein), biotin [77], or photoreactive tags such as trifluoromethyl diazirine, and 6-nitroveratryl alcohol, as very recently reported by Warminski et al. [78]. Similarly, azido-functionalized analogues with Cy3 or Cy5 florescent dyes, were reported [79]. The 2′-propagyl substituted cap analogue was also the subject of fluorescent labelling (with Alexa Fluor) [69] (Figure 6).

**Figure 7:**
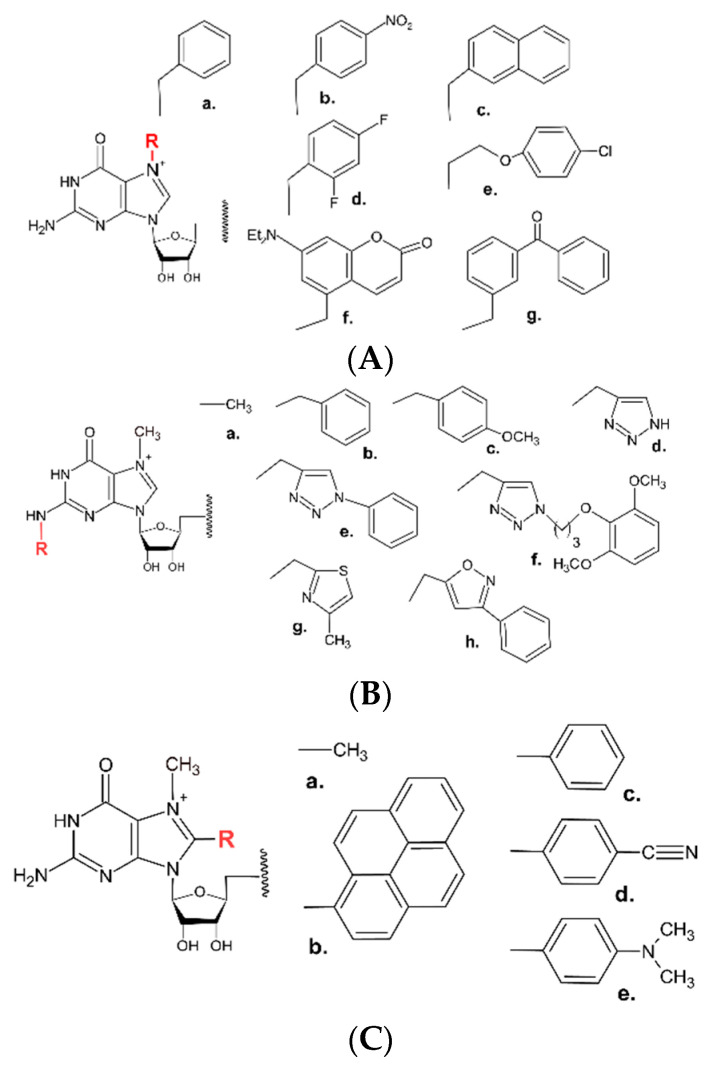
Examples of the modifications of N^7^, N^2^, and C8 sites of m^7^Guanine. (**A**) N^7^ substitutions include: (**a**) benzyl, (**b**) 4-nitrobenzyl, (**c**) naphtalene, (**d**) 2,4-difluorobenzyl, (**e**) 4-chlorophenoxyethyl, (**f**) 7-(diethylamino)-4-methyl-coumarin, (**g**) benzophenone. (**B**) N^2^ substitutions include: (**a**) methyl, (**b**) benzyl, (**c**) 4-methoxybenzyl, (**d**) triazole, (**e**) 1-phenyl-triazole, (**f**) 2,6-dimethoxyphenyl triazole, (**g**) 3-phenyl-isoxazole, (**h**) 4-methyl-thiazole. (**C**) **C8** substitutions include: (**a**) methyl, (**b**) 1-pyrene, (**c**) benzyl, (**d**) 4-dimethylaminophenyl, (**e**) 4-cyanophenyl.

Modifications of the cap structure at the N^7^, N^2^ or C8-position of the nucleobase of m^7^G have also been widely described. Initial focus was on the substitution of the N^7^ methyl moiety, as it is one of the structural features of the cap required for specific recognition by the eIF4E translation initiation factor, as revealed by crystallographic and biophysical data [80,81,82]. In turn, the N^7^-substituted compounds with diverse alkyl and aryl groups were synthesized [49,83,84,85]. Among those, N^7^-benzyl substitution increased inhibitory properties of mononucleotide analogues, and its pronucleotide counterpart appeared promising for targeting lung and breast cancer [39,49,86]. Furthermore, mRNA transcripts bearing N^7^-benzyl (or its derivatives) dinucleotide cap analogues showed higher translational activity [83,85,87]. Another favourable modification site was the N^2^ position of m^7^G, where the presence of a single methyl group has a positive effect on inhibition and translation [49,83]. Several other N^2^-alkyl- and -aryl-substituted analogues, including N^2^-benzyl and N^2^-(4-metoxybenzyl) ARCA, were next reported [88,89]. Further N^2^-modified analogues included triazole-, isoxazole-, and thiazole-ring-based substituents [10,90,91,92]. Combined modifications at N^2^ position of m^7^G and triphosphate bridge of dinucleotide and trinucleotide cap analogues (Cap 1–type) have also been described [93]. Recently, the synergistic effect that increase the inhibitory potency of synthesized compounds was shown for simultaneous modification at both N^7^ and N^2^ positions of m^7^G of mono- and dinucleotide analogs [41]. Photolabile groups put at the N^2^ (such as ortho-nitrobenzyl [94,95,96]) or at the N^7^ position (e.g., bezophenone or coumarin [97,98], represent yet another series of 7-methyl-guanosine modified cap analogues that can be incorporated into mRNA transcripts. Upon irradiation and photodeprotection of the cap structure, these analogues trigger the cap-dependent translation. Inagaki et al. [96] also synthesized cap analogues where the nitrobenzyl photocleavable tag was introduced at 2′-OH or 3′-OH position of m^7^G. Additionally, the m7G nucleobase of the cap structure can be modified at the C8-position, as demonstrated by Wojtczak et al. [99] (Figure 7).

Further development of synthetic cap analogues has led to the creation of trinucleotide and tetranucleotide compounds. Those, from one point of view, facilitated in vitro capping of mRNA transcripts in a correct orientation (such as ARCA analogues [9]; from another perspective they allowed the acquisition of analogues that reproduce the Cap-1-type and the Cap-2-type structures, that are present on mRNA transcripts in higher eukaryotes, including mammals [8,37], that are involved in suppression of the innate response activation trough IFIT1 and RIG-I [100,101,102] and confer resistance towards DXO activity [103]. Following the report of a series of m^7^GpppApG trinucleotide cap analogues (with differentially methylated adenosine: A, A_m_, m^6^A, m^6^A_m_) [18] and commercially available CleanCap trinucleotides [104], the trinucleotide analogues with the remaining nucleobases (in relation adenosine) were synthesized (m^7^GpppNpG, where N = C,C_m_, U, U_m_, G, G_m_) [105], as were also the tetranucleotide cap analogues (with Cap 2 signature) [103]. Additionally, other tri- and tetranucleotide cap analogues with modifications within the triphosphate bridge and/or m^7^G have been described [65,69,74,93,96,106,107].

Following the discovery of non-canonical cap structures, the synthesis of their analogs (both di- and trinucleotide series, with additional modification within the phosphate linkage) represents yet another series of compounds that facilitate the in vitro synthesis of NAD-, FAD-, or UDP-glucose-capped RNA transcripts [108,109,110].

## 4. NUDIX Protein Family

NUDIX enzymes are evolutionary conserved family of pyrophosphohydrolases found across all domains of life, including viruses. NUDIX (namely NUcleoside DIphosphates linked to a moiety X) possess a broad range of substrate specificity, including canonical (d)NTPs, oxidized nucleotides, dinucleotide polyphosphates, nucleotide sugars, capped RNAs, cap structures, and dinucleotide coenzymes (NAD, FAD, dpCoA) and their derivatives [111,112]. The *E. coli* MutT protein is referred to as a founding member of this superfamily, that is characterized by a highly conserved NUDIX motif (GX_5_EX_5_[UA]XREX_2_EEXGU, where U is hydrophobic and X any amino acid) [111,113]. The glutamic acid residues within the NUDIX motif (REUXEE) play role in the binding of divalent metal ions required for the catalytic activity of NUDIX enzymes [111]. The mechanism of substrate hydrolysis by NUDIX enzymes proceeds, in most cases, through nucleophilic substitution by water molecule at specific phosphorus of a di-(or polyphosphate) chain, but hydrolysis can occur also by nucleophilic substitution at carbon as it is in the case of the GDP-mannose hydrolase NUDIX subfamily [114]. Available data (based on crystal structures, mechanistic and kinetic studies) have indicated that NUDIX hydrolases usually require two or three metal ions for catalysis [114,115]. A recent study that used time-resolved X-ray crystalography of MutT, with its substrate (8-oxo-dGTP) and Mn^2+^, allowed for detailed visualization of the reaction process that proceed through nucleophilic substitution by water molecule with three Mn^2+^ ions with captured structures of intermediate reaction states [115].

Among eukaryotic NUDIX hydrolases, Dcp2 (Nudt20) was the first identified enzyme involved in removing the cap structure present at the 5′ end of mRNA [11,12,13,14]. An additional set of seven NUDIX mammalian enzymes possess mRNA cap hydrolysis activity in vitro (Nudt2, Nudt3, Nudt12, Nudt15, Nudt16, Nudt17 and Nudt19 [116,117]) and in vivo (Nudt3 and Nudt16 [15,118,119]). Aforementioned NUDIX proteins cleave within the triphosphate bridge of a cap structure, between α-β, β-γ or both α-β/β-γ phosphates (Figure 1) (e.g., [117]). Enzymatic activity of these NUDIX-decapping enzymes is influenced by the presence of specific modifications in the cap structure (e.g., by the presence of 7-methylganosie or methylation of adenosine adjacent to m^7^G for hDcp2 activity [14,19]; by the nucleobase type, as caps with adenosine (or 2′-O adenosine) are not subject to effective hydrolysis with hNudt16 [120,121]; or by the preference of hNutd16 for unmethylated cap [120]. As all these sites of the mRNA cap structure are subject of intensive/plethora chemical modifications in order to optimize its effect on mRNA stability, translation efficiency, and immunogenicity (Figure 4), the NUDIX proteins with different specificities towards cap structure(s) emerge as a powerful tool in their analysis.

## 5. RNA Decapping with Mammalian Nudt2, Nudt5, Nudt12, Nudt16, and Dcp2

In the case of the in-vitro-transcribed mRNA (IVT-mRNA), the presence of the cap structure at the 5′ end is one of the key features that enhances its translational activity and stability, thus underpinning the development of mRNA-based therapeutics. As the Dcp2 (Nudt20) is the major decapping enzyme in the 5′- to -3′ mRNA degradation pathway [122], recombinant hDcp2 is used in the in vitro assays to test stability of RNA transcripts capped with modified cap analogues or with analogues of the Cap 1 and Cap 2 structures (e.g., [55,56,83,103,105]). Dcp2 interacts with several regulatory proteins that act as decapping activators, including Dcp1 (*S. pombe* Dcp1 enhanced the Dcp2 catalytic activity 10-fold in vitro [123] or PNRC2 that, together with Dcp1, synergistically stimulates decapping activity of hDcp2 [124]. Both mentioned Dcp2 protein complexes were used in decapping susceptibility analysis of in vitro capped RNA: with N2-modified caps (here *Sp*Dcp1/Dcp2complex [89] or with N6-benzylated cap analogues (here: PNRC2-hDcp1/Dcp2 complex [107]. With regards to in-vitro-designed therapeutic (m)RNAs, next to the principal confirmation of enzymatic resistance of modified-cap –RNAs, a few additional aspects regarding Dcp2-mediated decapping should be taken into account. First, Dcp2 is not ubiquitously present throughout different human tissues [119]. Second, it is not involved in bulk mRNA decay in cells but rather is important for specific degradation pathways (e.g., nonsense-mediated decay, microRNA-mediated mRNA decay, or degradation in response to interferon [125,126]. Third, methylation status of adenosine adjacent to 7-methylguanosine in the cap structure (m^7^Gpppm^6^A_m_) was shown to be involved in decreased susceptibility to Dcp2 decapping [19] and that this phenomenon is related to selected transcripts rather than to general mRNA stabilization [105,127] (Table 2).

hNudt16 is another NUDIX enzyme used in analysis of modified-cap –RNA transcripts. It was initially identified as U8 snoRNA binding protein with decapping activity, which was shown for in vitro m^7^G- and m_3_^2,2,7^G- capped RNA [15]. Further studies revealed that it is ubiquitously detected in all analysed human tissues, is responsible for decapping at least of a subset of mRNA in cells [119] and is able to hydrolyse m^7^G-capped RNA transcripts in vitro [119,128]. Recombinant hNudt16 appeared as a potent tool to test triphosphate bridge modification of trimethylated m^7^G cap analogues and capped RNAs in a study on m_3_^2,2,7^G cap as a nuclear import signal and m_3_^2,2,7^G-mediated transport of therapeutics [121,129]. A direct comparative analysis pointed out to non-methylated cap structure at the 5′ RNA transcript end (GpppG-, GpppA-) as preferred substrate for hNudt16 decapping [120] and that decapping of RNAs with mono- and trimethylated cap structures is influenced by the nucleotide type adjacent to m^7^G (or m_3_^2,2,7^G)—those containing adenosine (or 2′-O adenosine) are very poor substrates to hNudt16 [120,121] (Table 2). This is in line with hNudt16 substrate specificity towards m^7^G dinucleotide cap analogues, where m^7^GpppA, m^7^GpppA_m_, and m^7^Gpppm^6^A_m_ appeared resistant to hNudt16 hydrolysis [130]. Additionally, modification of guanosine moiety (but not m^7^G) at the C8-position in the dinucleotide compounds resulted in their resistance to hNudt16 (except for the pyrene substituent) [99]. hNudt16 preference to the exposed unmethylated guanosine in those C8-modified cap analogues was shown also for dinucleotide diphosphate compounds, where the methylated m^7^GppG was hydrolysed around eight-fold slower in comparison to GppG [131]. This specific hNudt16 feature was used in developing of the “cap-orientation” test in analysis of RNA transcripts with N2-susbstituted caps [92]. Here, the GpppG-capped transcript underwent complete decapping with hNudt16, whereas m^7^GpppG-RNA SP6 polymerase transcript (that is a mixture of correctly and in-reverse-orientation capped products, m^7^GpppG-RNA and GpppGm^7^-RNA) was decapped at around 45%. In the correct orientation, ARCA-capped transcript was decapped at around 8% in the same experimental conditions, which is attributed to the lower activity of hNudt16 towards m^7^G-capped RNA [92,120]. Functionality of that test was shown for mRNA transcripts capped in vitro with a series of N^2^-modified dinucleotide cap analogues (triazole, isoxazole or thiazole substituted) [92,93]. RNA transcripts bearing cap structure with the attached FAM moitety at 2′-OH or 3′-OH of m^7^G were also subject to decapping with hNudt16 [77]. Next to the aforementioned analogues of the canonical cap structure, hNudt16 showed enzymatic activity towards RNA capped with non-canonical metabolite 5′ caps (NAD, dpCoA and FAD) [132]. Such wide activity of hNudt16 towards different 5′ cap structures on in vitro prepared RNA transcripts points out to it as a convenient, single enzyme that can be widely used in testing functionality of introduced chemical modifications within a polyphosphate linkage or within a nucleotide part of cap structure, and in test of cap-orientation and capping efficiency of non-ARCA cap analogues. It can be also considered in removing of the incorrectly (reversely) capped RNA transcripts after transcription in vitro with RNA polymerase. In such a case, hNudt16 treatment would expose monophosphate at the 5′ end of RNA (e.g., pGm^7^-RNA, after GpppGm^7^-RNA decapping), which is then degraded by Xrn1 processesive 5′ to 3′ exoribonuclease (similarly to removing of 5′ triphosphorylated RNA from the primary transcripts after 5′RNA polyphosphatase treatment, e.g., [107]).

Mammalian Nudt12 NUDIX enzyme was also shown to possess decapping activity of m^7^GpppG- and GpppG-capped transcripts in vitro [116]. Subsequent reports identified human and murine Nudt12 as a cytosolic RNA deNADing enzyme [133,134] and its decapping activity towards dpCoA-capped RNA [132]. Trimethylated cap structure at the 5′ end of RNA has been also effectively hydrolyzed with Nudt12, similarly to unmethylated an monomethylated caps [130]. Studies on differentially methylated dinucleotide cap analogs pointed out towards compounds containing adenosine (with different methylation status, e.g., A, A_m_ or m^6^A_m_) as preferred substrates for Nudt12, which is different in comparison to Nudt16 activity. For example, in the case of trimethylated analogues (m_3_^2,2,7^GpppG and m_3_^2,2,7^GpppA), hNudt12 efficiently hydrolysed dinucleotides with adenine but not guanine, whereas hNudt16 showed the opposite effect [130]. Thus, it seems reasonable to replace Nudt16 with Nudt12 in functional decapping studies of RNA transcripts modified cap analogues with adenosine as a nucleoside adjacent to initial m^7^G, as deNADing enzyme Nudt12 was already used in decapping test of RNAs with synthetic NAD analogues modified within diphosphate bridge [108].

Nudt2 was among other mammalian NUDIX enzymes with decapping activity towards canonical cap and metabolite FAD-cap and dpCoA cap [116,132]. In a recent report, following the discovery of diadenosine tetraphosphate capped RNAs, Nudt2 was shown to be responsible for the hydrolysis of AppppA-RNA [33]. Extended data for in vitro Nudt2-mediated decapping of Ap_n_A-RNA, Gp_n_G-RNA, Ap_n_G-RNA, and Gp_n_A-RNA showed its primary preference towards Ap_n_A-capped substrates (Table 2). Among other dinucleotide structures those with tetraphosphate bridge were also effectively hydrolyzed. Interestingly, Ap_3_G-RNA appeared resistant to Nudt2 decapping, opposite to Gp_3_A-RNA [33]. Interestingly, Nudt2 hydrolase triphosphorylated pppN-RNA into monophosporylated pN-RNA, which serves as a substrate for Xrn1 exonuclease [135] and as such could be considered as another enzymatic activity that can be used in removing uncapped pppN-RNA transcripts, similarly to the earlier-mentioned approach that utilizes commercially available 5′ polyphosphatase.

**Table 2:** Comparison of decapping activity of Dcp2, Nud16, Nudt12, Nudt2 towards RNA transcripts with indicated chemically modified and natural 5′ cap structures.

	Dcp2	Nudt16	Nudt12	Nudt2
m^7^GpppN	+ [105]			
m^7^GpppN_m_	+ [105]			
m^7^GpppG	+ [92,105,120,136]	+/− [92,93,120]	+ [116,130]	+ [116]
m^7^GpppA	+ [105]	− [120]		
m^7^GpppG_m_	+ [105]	+/− [120]		
m^7^GpppA_m_	+ [105]	− [120]		
m^7^Gppp^m6^A_m_	+ [105,107] − [19] (transcript dependent)			
m^7^GpppA_m_G_m_	+ [103]			
m^7^Gppp^bn6^A_m_G	+ [107]			
GpppG	− [14]	+ [92,93,120]	+ [116,130]	+ [33,116]
GpppA		+ [120]		+ [33]
ApppA				+ [33]
ApppG				− [33]
GppppG				+ [33]
GppppA				+ [33]
AppppA				+ [33]
AppppG				+ [33]
m_2_^7,3′O^GpppG	+ [58,89,136]	− [92,93,120]		
m_2_^7,2′O^GpppG	+ [38]			
m_2_^7,2′O^GppppG	+ [136]			
m_2_^7,2′-O^GppCH_2_ppG	− [136]			
m_2_^7,2′-O^GppCCl_2_ppG	− [136]			
m_2_^7,2′-O^GppCF_2_ppG	− [136]			
m_2_^7,2′-O^Gpp-tz-C_2_H_4_OppG	− [64]			
m_2_^7,2′-O^Gppp-tz-C_2_H_4_OpG	+ [64]			
m_2_^7,2′O^GppNHpN	− [56]			
m_2_^7,3′O^GppCH_2_pN	− [56,58]			
m_2_^7,2′O^Gpp_S_pG (D1)	−/+ [38]			
m_2_^7,2′O^Gpp_S_pG (D2)	−/+ [38]			
m_2_^7,2′O^Gpp_BH3_pG (D2)	− [38]			
bn^2^m^7^GpppG	+ [92]	−/+ [92]		
bn^2^m^7^Gpp_S_pG		− [93]		
bn^2^m_2_^7,3′O^GpppG	+ [89]			
bn^2^m_2_^7,2′O^GpppG	+ [89]			
bn^2^m^7^GppppG		+ [93]		
bn^2^m^7^GpppA_m_G		− [93]		
(4-Cl-bn)^2^m^7^GpppG	+ [92]	−/+ [92]		
(4-Cl-bn)^2^m^7^GppppG		+ [93]		
(4-Cl-bn)^2^m^7^GpppA_m_G		− [93]		
(4-OCH_3_-bn)^2^m^7^GpppG	+ [92]	−/+ [92]		
(4-OCH_3_-bn)^2^m_2_^7,3′O^GpppN	+ [89]			
(4-di(OCH_3_-bn)-tz)^2^m^7^GpppG	+ [92]	− [92]		
(4-di(OCH_3_-bn)-tz-CH_2_)^2^m^7^GpppG		− [93]		
(4-di(OCH_3_-bn)-tz-(CH_2_)_2_)^2^m^7^GpppG		+/− [93]		
(4-di(OCH_3_-bn)-tz-(CH_2_)_4_)^2^m^7^GpppG		− [93]		
(4-bn-isx)^2^m^7^GpppG	+ [92]	−/+ [92]		
(4-CH_3_-th)^2^m^7^GpppG	+ [92]	−/+ [92]		
2′-O/3′-O-(FAM-L6N)-m^7^GpppG	+ [77]	+ [77]		
m_3_^2,2,7^GpppG		+ [120,129]	+ [130]	
m_3_^2,2,7^GpppA		−/+ [121]		

Human Nudt5 is the NUDIX hydrolase that has been linked to nucleotide metabolism and cancer [137]. Nudt5 did not show decapping activity towards m^7^GpppG- and GpppG-capped RNAs [116]. Recently, for the recombinant hNudt5, it showed decapping activity towards non-canonical UDP-GlcNAc cap on RNA transcripts in vitro [110] (Table 3). Thus, it could be a potential tool to analysis of UDP-glucose-derived cap synthetic analogues for future investigation of this 5′ end RNA modification in mammalian cells [37].

## 6. Other RNA Decapping Enzymes

Decapping of (m)RNA cap structures was shown in vitro for NUDIX enzymes in other domains of life—in plants [138], bacteria, or viruses [139]. Among four tested enzymes of the model plant *Arabidopsis thaliana*, the AtNUDT27 appeared as effective enzymes with a broad activity towards various cap structures: unmethylated, monomethylated m^7^G-caps, and non-canonical caps [138]. RppH bacterial NUDIX hydrolase was initially identified as activity that hydrolyzes 5′-triphoshate from RNA leaving 5′-monophosphate RNA [140], and subsequently it was demonstrated to cleave m^7^GpppG- and GpppG-capped RNA [116] and NAD-capped transcripts [133]. deNADing activity was also attributed to bacterial NudC enzyme [141].

Apart from the NUDIX-type decapping enzymes, at least three other classes of enzymes are involved in processing cap structures at the 5′–RNA end: DXO family proteins, HIT (histidine triad) proteins, and ApaH-like phosphatase found in trypanosomes [139]. In the case of DXO, it removes cap structure through cleavage of the entire dinucleotide from the RNA body (e.g., GpppG, m^7^GpppG, m_3_^2,2,7^GpppG), with a preference towards unmethylated structure, which was connected to functions in the mechanism of 5′cap quality control [142]. 2′O-methylation of the first and the second transcribed nucleotide found in the Cap 1 and Cap 2 structures of mRNA, respectively, caused resistance to DXO decapping [101,143]. Apart from decapping activity, DXO can also remove non-canonical caps (NAD, FAD, and dpCoA) and has activity toward uncapped triphosphorylated RNA [144].

## 7. Discussion

Among mammalian NUDIX hydrolases with (m)RNA decapping in vitro, Dcp2 and Nudt16 have been used in the analysis of diverse analogues of canonical cap structure. However, mRNA decapping by Dcp2 is a complex process involving multiple regulatory proteins, such as Dcp1, Edc3, and Edc4. The formation of various Dcp2-containing multiprotein complexes ensures precise control over mRNA stability and degradation in vivo, which is crucial for cellular homeostasis and response to environmental changes [145,146]. Moreover, as mentioned earlier, Dcp2 controls stability only of a subset of mRNA in cells [115], exemplified by the recent global profiling study that revealed a number of Dcp2 mRNA substrates, including innate immunity-related transcripts [147,148]. Thus, for the more physiologically relevant results, in vitro decapping with Dcp2 should also be reconsidered in terms of the decapping complex with regulatory proteins and designed gene-specific mRNA (e.g., involved in innate immunity).

Available evidence points out to Nudt16 as a decapping enzyme with activity towards the widest range of different cap structures at 5′ end of RNA transcripts: umethylated (GpppN-), mono-methylated (m^7^GpppN-), trimethylated (m_3_^2,2,7^GpppN-), NAD-, FAD-, and dpCoA-cap. Bearing in mind the broad substrate specificity, Nudt16 can be considered as the novel major standard in analysis of modified cap structures at (m)RNA transcripts produced in vitro, next to Dcp2. It already proved to be a potent tool in studies of caps modified in a triphosphate bridge and within 7-methylguanosine (N^2^ and 2′-OH or 3′-OH of m^7^G). Additionally, it could be used in cap-orientation assay and removing of the reversely capped (with non-ARCA cap analogues) RNA transcripts from IVT reactions (when combined with Xrn1 exoribonuclease). Important advantage of Nudt16 is that it acts effectively as a single subunit, without additional regulatory proteins, what can facilitate its use and optimization. One of the possible limitations is its use in regards to transcripts bearing at their 5′ end dinucleotide cap analogues with adenosine adjacent to the m^7^G (or m_3_^2,2,7^G), which are poor substrates for Nudt16 decapping. However, the Nudt12 hydrolase could be used in such case instead. Finally, Nudt2 enzyme is active in vitro towards unmethylated Np_n_N-RNAs (where N = G or A), with lower activity towards those with triphosphate bridge (Np_3_N-RNAs) in comparison to di- or tetraphosphate-containing dinucleotide structures. As such, Nudt2 can be an attractive tool in testing modifications introduced into the polyphosphate chain of synthetic non-methylated dinucleotide analogues (e.g., GppXpG). Those in turn, after incorporation into (m)RNA during IVT reaction, could be subject to methylation by guanine-7-methyltranferase specific activity (e.g., Vaccina Capping Enzyme, VCE) or introduction, e.g., benzylic moieties with in-vitro-engineered MAT/MTase (methionine adenosylotrasferase/methyltransferse) activities [87]. Nudt2 also showed in vitro activity to m^7^GpppN-capped transcripts. Thus, it cannot be excluded from analysis of methylated cap structures. However, further characterization with a wider set of such analogues is needed in that context. Another Nudt2 activity of potentially great interest is its ability to convert triphosphorylated pppN-RNA into monophosporylated pN-RNA, the latter being a substrate for Xrn1-riboexonuclease-mediated removal from IVT reaction. Among identified mammalian NUDIX proteins with in vitro decapping activity towards canonical methylated and unmethylated cap structures are four additional enzymes: Nudt3, Nudt15, Nud17, and Nudt19 [116,117]. These represent an as-yet-unexplored field in the study of decapping of RNAs with modified cap analogues. As Nudt3 was shown also to be active in vivo toward a subset of mRNAs involved in cell migration regulation [118], it can be considered, next to the Nudt proteins described above, in future assay(s) aimed at the stability of RNA transcripts.

## 8. Conclusions

Next to the Dcp2, at least three other NUDIX enzymes were reviewed here—Nudt16, Nudt12, and Nudt2 comprise a set of activities that can be used in the 5′ capped RNA transcripts analysis that bears a diverse chemical modifications introduced into 5′ cap structure. They can be also considered in preparation of homogenous RNAs as a result of removal of incorrectly capped or 5′ triphosphorylated transcripts, or preparation of 5′ RNA end for downstream methodologies (e.g., CapZyme-Seq [149]). Given the known differences in the specificity of Nudt16, Nudt12, Nudt2, and Dcp2 towards natural and modified cap structures, the combinatorial use of these enzymes of human origin as a “NUDIX decapping toolbox” seems more relevant in the context of further development and use of capped mRNAs as a therapeutic molecule and their translation into the clinic. Finally, as they act effectively without additional activatory proteins, this facilitates their use and optimization.

## Figures and Tables

**Table 3:** Activity of selected NUDIX proteins towards metabolite non-canonical RNA caps.

	NAD Cap	FAD Cap	dpCoA Cap	UDP-GlcNAc Cap
Nudt16	+	+	+	
Nudt12	+		+	
Nudt5				+
Nudt2		+	+	
